# Vitamin C Intake and Ischemic Stroke

**DOI:** 10.3389/fnut.2022.935991

**Published:** 2022-07-14

**Authors:** Xiaolong Tang, Hanguang Liu, Yuan Xiao, Lei Wu, Peng Shu

**Affiliations:** ^1^Department of Internal Neurology, Beilun District People's Hospital, Ningbo, China; ^2^Department of Painology, The No. 1 People's Hospital of Ningbo, Ningbo, China; ^3^Department of Molecular Laboratory, Beilun District People's Hospital, Ningbo, China

**Keywords:** vitamin C, ischemic stroke, review, micronutrient, CVD

## Abstract

Vitamin C is an essential micronutrient with important antioxidant properties. Ischemic stroke is a major public health problem worldwide. Extensive evidence demonstrates that vitamin C has protective effects against cardiovascular disease, and there is a close relationship between vitamin C intake and ischemic stroke risk. Based on the evidence, we conducted this umbrella review to clarify the relationship between vitamin C intake and ischemic stroke risk from four perspectives: cellular mechanisms, animal experiments, clinical trials, and cohort studies.

## Introduction

Stroke is the second most common cause of death and the leading cause of disability and, therefore, a major public health concern (1). Stroke is associated with high rates of morbidity, disability, mortality, and recurrence (1). Ischemic stroke is the most common type of stroke, accounting for approximately 80% of all cases of stroke (1). Although the mortality rate of stroke has decreased globally in recent years, the global disease burden of stroke has continued to increase; thus, the prevention and treatment of stroke are important components of global public health management (1). Vitamin C is an essential nutrient with antioxidant and anti-inflammatory properties, and has been shown to inhibit the inflammatory response and oxidative reactions, protect the vascular endothelium, and prevent the development of atherosclerosis (2). This review systematically discusses the relationship between vitamin C intake and ischemic stroke risk from four perspectives: cellular mechanisms, animal experiments, clinical trials, and cohort studies.

Vitamin C, also known as ascorbic acid, cannot be synthesized by the human body and must be obtained through the diet (3). It is a water-soluble vitamin that is present in a wide range of fruits and vegetables. As an essential micronutrient in the human body, an adequate intake of vitamin C helps maintain human health (3). Vitamin C is a water-soluble acidic polyhydroxy compound with six carbon atoms and a structure similar to glucose (4). It has many biological functions. It produces H^+^ after being oxidized to dehydrovitamin C. The oxidized and reduced forms of vitamin C can be converted into each other to form a redox system in biological tissues (4). Many physiological activities of vitamin C are related to this property. For instance, vitamin C functions as a coenzyme or a substrate for a series of enzymes involved in various metabolic pathways: it is a coenzyme for prolyl and lysyl hydroxylases, which catalyze the hydroxylation of proline and lysine, respectively, during collagen biosynthesis, and a coenzyme in iron metabolism (4).

The circulating vitamin C concentration in healthy people is approximately 70 μM. Concentrations below 23 μM indicate vitamin C deficiency, and concentrations below 11 μM indicate severe vitamin C deficiency with a risk of scurvy (5). The amount of vitamin C intake required by the human body depends on the plasma ascorbic acid concentration (5). While the recommended dietary allowances (RDAs) provide estimates of the required vitamin C intake for humans, the optimal dietary intake is unknown and may be determined by factors such as the dose–function relationship, the availability of vitamin C in dietary sources, the plasma and tissue steady-state concentrations after each dose of vitamin C, urinary excretion, bioavailability, toxicity, and epidemiological observations of vitamin C intake (5). The relationship between the plasma vitamin C concentration and intake dose shows an S-shaped curve (5). Plasma vitamin C concentrations of 50 μM and higher are considered to be appropriate. The RDA of vitamin C varies widely among different health organizations. The German, Austrian, and Swiss Institutes of Nutrition have stipulated an RDA of 110 mg/day for men and 95 mg/day for women, whereas the American and Canadian Institutes of Medical Research have set an RDA of 90 mg/day for men and 75 mg/day for women (6, 7).

## The Possible Mechanism Whereby Vitamin C Reduces the Risk of Ischemic Stroke

Vitamin C may reduce the risk of ischemic stroke through various mechanisms, such as inhibiting low-density lipoprotein (LDL) oxidation, increasing intravascular nitric oxide (NO) production, increasing vasodilation and lowering blood pressure, and reducing the adhesion of monocytes to the vascular endothelium, thereby reducing atherosclerosis (8, 9).

### Vitamin C Inhibits the Inflammatory Response

Vitamin C has strong reducing properties due to it being a strong antioxidant. In the human body, it can inhibit the formation of oxygen free radicals, regulate inflammatory factors, inhibit inflammatory cell infiltration, reverse endothelial dysfunction, improve microcirculation, and alleviate the micro-inflammatory state (10). Mohammed et al. (11) found that vitamin C-sufficient mouse macrophages exhibited an obvious anti-inflammatory phenotype, whereas vitamin C-deficient mouse macrophages continued to express interleukin (IL)-1 (*IL-1*), tumor necrosis alpha (*TNF-*α), and monocyte chemoattractant protein-1 mRNAs, indicating a pro-inflammatory phenotype. Block et al. (12) found that the serum C-reactive protein concentrations were significantly reduced in active and passive smokers after oral administration of vitamin C. Mikirova et al. (13) found that the intravenous injection of vitamin C in cancer patients resulted in significant decreases in the serum concentrations of the inflammatory cytokines IL-1α, IL-2, IL-8, and TNF-α; the eosinophil chemokine eotaxin; and C-reactive protein.

**Figure 1:**
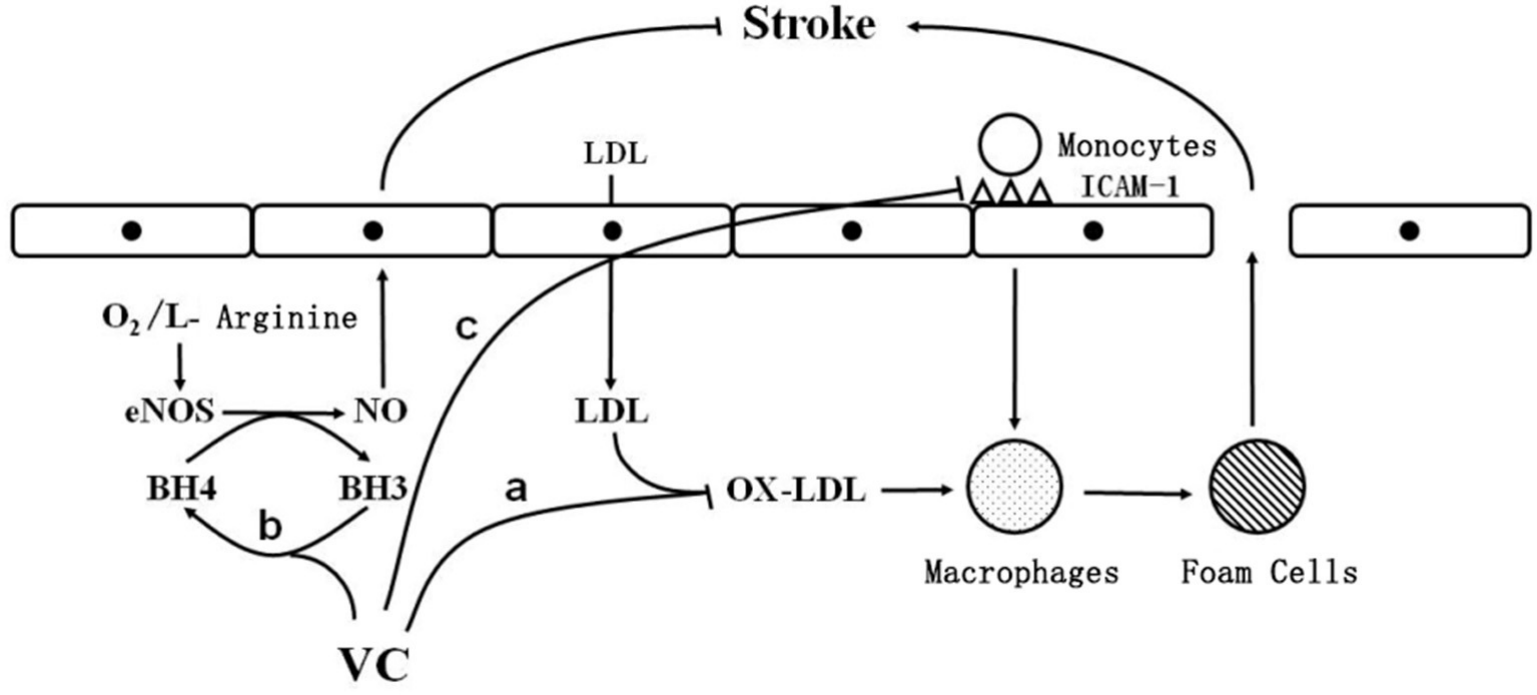
The cellular mechanism by which vitamin C reduces the risk of ischemic stroke. O_2_: oxygen; eNOS: endothelial nitric oxide synthase; BH3: trihydrobiopterin; BH4: tetrahydrobiopterin; vc: vitamin C; NO: nitric oxide; LDL: low density lipoprotein; OX-LDL: oxidized low density lipoprotein; ICAM-1: intercellular adhesion molecule-1; stroke: stroke; a: vitamin C inhibits low-density lipoprotein oxidation; b: vitamin C reduces trihydrobiopterin to tetrahydrobiopterin; c: Vitamin C reduces the expression of ICAM-1.

Atherosclerotic plaques are important risk factors for cerebrovascular disease (14). Activation of the inflammatory response reduces the stability of a plaque, leading to its rupture (14). Importantly, secondary thrombosis and embolism are the main mechanisms of ischemic stroke (15). Early in the formation of atherosclerotic plaques, monocytes adhere to the endothelial wall, causing the vessel wall to thicken and lose its elasticity (15). Vitamin C has been found to reduce the adhesion of monocytes to the vascular endothelium (8, 9) by decreasing the expression of intercellular adhesion molecule-1, a surface glycoprotein that mediates the adhesion of monocytes to endothelial cells (16) (Figure 1).

### Vitamin C Inhibits Oxidative Reactions

Studies have shown that oxidative stress and its related molecular events play important roles in the pathological process of ischemic stroke (17). Ischemic stroke occurs due to a sudden interruption of the cerebral arterial blood supply due to the occlusion of cerebral arteries, which in turn leads to cerebral hypoxia and the accumulation of reactive oxygen species (18). When blood flow is restored, oxidative stress in the brain may be exacerbated, leading to an imbalance between the production of oxidants and the antioxidant defense mechanisms, resulting in dysregulated cell survival mechanisms and, ultimately, nerve damage (18).

Under normal conditions, the production and elimination of free radicals in the body are balanced. When there are too many free radicals, cholesterol in lipoproteins, especially LDL, easily undergoes peroxidation, which is a risk factor for atherosclerosis and ischemic stroke (19). In addition, oxidized LDL is highly cytotoxic and can accelerate the formation of fatty streaks (20). Monocytes adhered to the endothelium are activated to differentiate into macrophages, which ingest large amounts of oxidized LDL, become enriched in cholesterol, and transform into foam cells, leading to the development of fatty streaks, thereby promoting the development of atherosclerosis (21, 22). Importantly, vitamin C inhibits LDL oxidation (Figure 1).

### Pro-oxidative Effects of Vitamin C

Regarding the pro-oxidative effect of vitamin C, the most intuitive evidence was obtained from the experiment performed by Griffiths et al. (23). Their results showed that U937 monocytes displayed increased production of reactive oxygen species after they were co-incubated with 150 μmol/L ascorbic acid and dihydrochlorofluorescein for 40 min.

The reason for the pro-oxidative effect of vitamin C has not been determined. Some scholars believe that this effect may be the result of the interactions between vitamin C and some metal ions (such as Fe^3+^). In 1996, Andorn et al. (24)confirmed that vitamin C can cause lipid peroxidation in the human brain, and this effect depends on the participation of iron ions, with iron at 100 mg·d^−1^ being able to cause uncontrollable lipid peroxidation. Similarly, Lachili et al. (25) found uncontrollable lipid peroxidation in pregnant women taking concurrent vitamin C (500 mg·d^−1^) and iron (100 mg·d^−1^) supplements.

In addition, some scholars believe that the pro-oxidative effect of vitamin C may be related to the ascorbic acid cycle. In this cycle, the dehydroascorbic acid transported into the cell is rapidly converted into ascorbic acid by enzymatic or non-enzymatic catalysis, and the resulting ascorbic acid causes the oxidation of other substances in the cell (23, 26). The oxidation of cellular substances was confirmed by Song et al. through a series of experiments in which the transport of dehydroascorbic acid was blocked with wortmannin (a glucose carrier-specific blocker), which reduced the vitamin C-induced production of lipid peroxidation products (26).

### Vitamin C Protects the Vascular Endothelium

Vitamin C reduces the inflammatory response by protecting against endothelial dysfunction via many mechanisms, including scavenging oxygen free radicals and inducing the synthesis of NO. Levine et al. (27) found that vitamin C reverses vascular endothelial dysfunction in patients with coronary heart disease. Animal experiments (28) have shown that vitamin C stabilizes tetrahydrobiopterin without dehydrogenation to allow endothelium-derived NO synthase to remain in a normal coupled state and maintain its normal activity. Cell culture experiments (29) have shown that vitamin C increases endothelium-dependent vasodilation by increasing the glutathione concentration in endothelial cells and inducing NO synthesis in these cells in a time- and dose-dependent manner.

Endothelial dysfunction is a main cause of ischemic stroke. After endothelial cell injury, platelet adhesion and aggregation accelerate thrombosis, leading to the development of ischemic stroke (30). Vitamin C stimulates endothelial cell proliferation by increasing the synthesis and deposition of type IV collagen in the basement membrane, thereby inhibiting apoptosis, and by stabilizing the NO produced by endothelial cells to regulate vascular tone and protect the vascular endothelium (31). Vitamin C reduces trihydrobiopterin radicals to tetrahydrobiopterin (Figure 1), which is an important cofactor for endothelial NO synthase (31). Tetrahydrobiopterin deficiency causes endothelial NO “uncoupling” and decreases NO production, leading to endothelial cell dysfunction (31). By maintaining the tetrahydrobiopterin concentration, vitamin C enables endothelial cells to produce normal amounts of NO, thus protecting vascular endothelial cells from damage, inhibiting the development of atherosclerosis, and reducing the risk of ischemic stroke (31).

## Animal Experiments on the Effect of Vitamin C on Ischemic Stroke

Yan et al. showed that simultaneous tetrahydrobiopterin, L-arginine, and vitamin C supplementation increased vascular perfusion after ischemia by increasing endothelial NO synthase activity and reducing oxidative stress (32). Through experiments using mice, D'Uscio et al. showed that vitamin C preserved vascular endothelial function by protecting tetrahydrobiopterin and restoring endothelial NO synthase activity (28). Using the Rice–Vannucci model, Miura et al. found that, in immature rats, intracerebroventricular injection of ascorbic acid after hypoxia–ischemia had neuroprotective effects; particularly, ascorbic acid inhibited cell necrosis and apoptosis in the brains of immature rats after hypoxia–ischemia-induced cell death (33, 34). Iwata et al. showed that during middle cerebral artery occlusion and reperfusion in rats with diabetes, ascorbic acid supplementation inhibited apoptosis and pro-inflammatory responses and alleviated brain injury and neurological deficits in the diabetic state (35). Furthermore, a study on patients with diabetes showed that ascorbic acid supplementation protected the endothelium from ischemia-induced oxidative damage (35). This is closely related to the reduction in intravascular reactive oxygen species levels mediated by ascorbic acid (36). These animal experiments show that vitamin C reduces the risk of ischemic stroke via antioxidant effects, thus protecting vascular endothelial function and inhibiting inflammation.

## Clinical Trials of Vitamin C in Ischemic Stroke

In recent years, there have been several randomized controlled clinical trials of vitamin C for ischemic stroke. Most of the experimental results have shown that vitamin C has no significant effect on reducing the risk of ischemic stroke (37–42). In a study in which 20,536 adults with coronary heart disease, other occlusive arterial disease, or diabetes were randomly assigned to receive vitamin C supplements or placebo, plasma vitamin C concentrations increased by one third in the supplement group during the 5-year intervention period, but there was no significant difference in stroke-related mortality between the two groups (41). In studies of populations with a high risk of stroke, vitamin C supplementation has shown no significant effect on stroke risk (41). In the American Men's Physician's Health Study, an intervention consisting of 400 IU of vitamin E every other day and 500 mg of vitamin C daily was associated with protective effects against cardiovascular disease compared with placebo after 8 years of follow-up (43). However, the occurrence of cardiovascular disease (CVD) was not significantly affected by the intervention, with the overall hazard ratio for stroke in the intervention group being 0.89 [95% confidence interval (CI), 0.74–1.07] (37). Similar results were reported in the Women's Antioxidant Cardiovascular Study, in which the intervention included 500 mg of vitamin C daily, 600 IU of vitamin E on alternate days, and 50 mg of beta-carotene on alternate days in women with a high risk of CVD (38). Vitamin C was found to have no overall effect on CVD or cerebrovascular events in these women (38). Studies by Blot et al., Hercberg et al., and Brown et al. found that vitamin C supplementation did not reduce the risk of stroke (39, 40, 42). These findings are consistent with the results of meta-analyses by Myung et al. and Ye et al. (18, 44). Lena et al. also found no evidence that vitamin C supplementation reduces the risk of stroke (21).

The design, endpoint, observation time, and study population of a clinical trial have important effects on the results. Accumulating evidence indicates that well-designed clinical trials are necessary to evaluate the effects of vitamin C on the risk of stroke and CVD (37–39). The greatest clinical benefit of vitamin C can only be achieved by designing more targeted clinical trials to evaluate its effect on CVD.

## Cohort Studies of Vitamin C and Ischemic Stroke

### Search Strategy for Systematic Review

This systematic umbrella review followed the Preferred Reporting Items for Systematic Reviews and Meta-Analyses (PRISMA) 2020 Statement guidelines (45, 46). Studies were identified through a comprehensive search of ProQuest, MEDLINE (PubMed), EBSCOhost, Web of Science, and ScienceDirect from the inception of the respective databases to June 2022. No language restrictions were applied. The search strategy included several MeSH terms: “Vitamin C” OR “micronutrient” OR “nutrients” AND “Ischemic Stroke” AND “Stroke”. The references cited in all of the eligible articles were also manually searched.

### Eligibility Criteria

Cohort studies evaluating vitamin C intake and ischemic stroke risk in humans were included. The inclusion criteria were studies that (1) included adults aged ≥18 years; (2) reported dietary vitamin C intakes or measured serum vitamin C levels; and (3) assessed the occurrence of ischemic stroke as the outcome.

### Study Selection and Data Collection

The selection of articles involved three stages: (1) title screening, (2) abstract screening, and (3) full-text review. Two investigators (XLT and HGL) screened the titles and abstracts independently and selected eligible articles through full-text review. Any discrepancies in selecting articles between the two researchers were resolved by a third investigator (LW).

Data extraction was performed using a data extraction table in which the following types of information were entered: (1) name of the first author, (2) journal, (3) publication year, (4) vitamin C intake, (5) outcome, (6) number of males and females, (7) number of participants in each study, (8) study design, (9) follow-up time, (10) type of comparison (highest vs. lowest intake of vitamin C), and (11) the estimated summary effect (relative risk) and corresponding 95% CIs.

Table 1 lists the cohort studies of vitamin C and ischemic stroke. Most epidemiological studies reported that vitamin C can reduce the risk of ischemic stroke. In Finland, 2,419 middle-aged men with no history of stroke were followed up for 10.4 years, and it was found that after adjusting for factors such as age, body mass index, smoking, and alcohol consumption, men with the highest plasma vitamin C concentration (64.96 μmol/L) had a reduced risk of stroke compared with men with the lowest plasma vitamin C concentration (28.40 μmol/L; hazard ratio for stroke: 0.48; 95% CI: 0.26–0.83), indicating that low plasma vitamin C concentrations are associated with an increased risk of stroke (53). A 20-year follow-up study in the United Kingdom confirmed that people with the lowest vitamin C status had the highest risk of stroke and that vitamin C concentrations in older adults were closely associated with stroke risk, regardless of whether vitamin C was measured in terms of plasma concentration or dietary intake (48). Similar findings were obtained in cohort studies in different countries (47, 52, 54–57, 59–61). However, some studies have reported inconsistent findings (49–51, 58).

**Table 1:** Cohort study of vitamin C and ischemic stroke.

**References**	**Research type**	**Time**	**Sample size (examples)**	**Age (years)**	**Vitamin C intake (mg/ day)**	**Follow-uptime (years)**	**Relative risk (95%CI)**	**Adjustment factor**
Gey et al. (47)	Observational (Measure vitamin C concentration in plasma)	Beginning: 1971–1973 Ending: 1985	2,974 Men	/	“Low””Normal”	12	0.24 (0.10–0.60)	Gender, smoking, blood pressure, cholesterol and beta carotene
Gale et al. (48)	Observational (7 days of dietary records, measurement of plasma vitamin C)	Beginning: 1973–1974 Deadline: Not mentioned	730 (equal number of men and women)	≥65	19.4 (T1) 53.4 (T3)	20	0.5 (0.3–0.8)	Age, sex and determined cardiovascular risk factors
Ascherio et al. (49)	Observational (Food Frequency Questionnaire)	1986–January 31, 1994	43,738 Men	40–75	95.00 (Q1) 1167.00 (Q5)	8	IS: 1.03 (0.66–1.59)	Age, smoking, hypertension, hypercholesterolemia, body mass index, physical activity
Hirvonen et al. (50)	Observational (dietary questionnaire)	Ending: 1993.4.30	26,593 Composition of male smokers	50–69	52.00 (F1) 141.00 (F4)	6.1	0.89 (0.72–1.09)	Age, BMI, blood pressure, cholesterol, height, smoking, history of diabetes or coronary heart disease, alcohol consumption and education
Yochum et al. (51)	Observational (semi-quantitative food frequency questionnaires, vitamin and mineral supplement intake)	1986–december 31, 1997	34,492 Postmenopausal women	55–69	82.40 (Q1) 678.70 (Q5)	11	1.23 (0.76–1.90)	Age, BMI, waist-to-hip ratio, smoking, diabetes, high blood pressure, physical activity, alcohol consumption, marital status and education level, intake of cholesterol, saturated fat, fish, dietary fiber, whole grains and other antioxidants
Yokoyama et al. (52)	Observational (Food Frequency Questionnaire)	1977–1997	2,121 (880 men and 1,241 women)	≥40	44.01 (F1) 52.13 (F4)	20	IS:0.71 (0.59–0.51)	Age, sex
Kurl et al. (53)	Observational (Measure vitamin C in plasma)	1984–1998.12.31	2,419 Middle-aged men	42–60	28.40 (T1) 64.96 (T4)	10.4	0.48 (0.26–0.83)	Age, BMI, systolic blood pressure, smoking, alcohol consumption, total serum cholesterol, diabetes, and exercise-induced myocardial ischemia
Voko et al. (54)	Observational (food frequency data)	1990 to 1993: before 1 January 1999	5,197 Men	≥55	T1 T3	6.4	0.66 (0.46–0.93)	Age, sex, total energy intake, smoking, hypertension, diabetes, coronary artery disease, history of TRANSIENT ischemic attack
Lee et al. (55)	Observational (Food Frequency Questionnaire)	January 1986–December 31, 2000	1,923 Postmenopausal women	55–69	85.00 (Q1) 667.00 (Q5)	15	1.89 (0.73–4.92)	Age, total energy intake, history of hypertension, BMI, waist-to-hip ratio, physical activity score, smoking, alcohol consumption, hormone replacement therapy, major type of diabetes medication use, and duration of diabetes
Myint et al. (56)	Observational (Health and Lifestyle Questionnaire (containing supplements or supplements containing vitamin C)	From 1993 to 1997 until March 2005	20,649 (Men 9,449 Women 11,200)	40–79	35.00 (Q1) 71.50 (Q5)	9.5	0.57 (0.43–0.76)	Age, sex, smoking status, BMI, systolic blood pressure, cholesterol, physical activity, myocardial infarction and diabetes mellitus
Del Rio et al. (57)	Observational (Semi-quantitative food Frequency Questionnaire)	From 1993 to 1998 to 31 December 2004	41,620 (Not mentioned)	44–61	83.00 (T1) 201.00 (T3)	7.9	IS:0.53 (0.31–0.89)	Age, sex, high blood pressure, smoking, education, alcohol consumption, waist circumference, BMI and total physical activity
Kubota et al. (58)	Observational (Semi-quantitative food Frequency Questionnaire)	From 1988 to 1990 to 2006	23,119 Men/35,611 Women	40–79	M:52.00 (Q1) 145.00 (Q5) F:60.00 (Q1) 150.00 (Q5)	16.5	M:0.84 (0.62–1.13) F:0.7 (0.54–0.92)	Age, history of hypertension and diabetes, smoking, alcohol consumption, body mass index, mental stress, physical activity, education level, total dietary energy intake, cholesterol, saturated fatty acids, n-3 fatty acids and sodium
Uesugi et al. (59)	Observational (Semi-quantitative food Frequency Questionnaire)	From 1995 to 1997–as of the end of 2009	82,044	45–74	60.00 (Q1) 239.00 (Q5)	15	0.76 (0.60–0.96)	Age, sex,BMI, smoking, alcohol consumption, physical activity, medication or history of diabetes, hyperlipidemia, and hypertension
Martín-Calvo et al. (60)	Observational (Semi-quantitative food Frequency Questionnaire)	Prior to March 2014–December 2016	13,421 (Not mentioned)	≥40	148.00 (T1) 445.00 (T3)	11	0.30 (0.12–0.72)	Gender,BMI, total energy intake, total fiber intake, physical activity, TV watching, smoking, cardiovascular disease, family history of stroke, and aspirin treatment
Lee et al. (61)	Observational (Semi-quantitative food Frequency Questionnaire)	Beginning 1995-1996- as of December 31, 2017	875	25–74	F1 F4	22	0.66 (0.52–0.85)	Age, sex, BMI, smoking, hypertension, dyslipidemia, abnormal blood glucose, and baseline history of cardiovascular disease

*IS: ischemic stroke; T1: the lowest third of vitamin C intake distribution; T3: the highest third of vitamin C intake distribution; F1: the lowest quartile of vitamin C intake distribution; F4: the highest quartile of vitamin C intake distribution; Q1: The lowest quintile of vitamin C intake distribution; Q5: Highest quintile of vitamin C intake; BMI: BODY mass index; CI: confidence interval; Relative risk: The highest quintile of vitamin C distribution compared with the lowest quintile*.

In an 8-year follow-up study, Ascherio et al. assessed the risk of stroke in the top and bottom quintiles (1,167 and 95 mg/day, respectively) of vitamin C intake among 43,738 men aged 40–75 years without CVD or diabetes. The relative risk of ischemic stroke in the top quintile was 1.03 (95% CI, 0.66–1.59) compared with the bottom quintile, and vitamin C supplementation did not significantly reduce ischemic stroke incidence in this cohort, which may be due to dietary measurement errors or the study subjects being medical professionals who had healthier lifestyles and eating habits than the average man (49). Yochum et al. concluded that vitamin C is not associated with stroke risk, which may be due to the pro-oxidative effect of vitamin C. Vitamin C not only is an antioxidant but also functions as a pro-oxidant in some cases (51, 62). Kubota et al. found that vitamin C was not associated with stroke risk in men, which may be due to the lower antioxidant capacity of dietary vitamin C or other risk factors for stroke in men (58). Hirvonen et al. concluded that the risk of stroke in their study subjects was probably attributable to smoking, and the influence of vitamin C on stroke risk may differ between smokers and non-smokers. Therefore, the results of their study cannot be generalized to non-smokers (50).

These inconsistencies between the results of different studies may be due to differences in the ethnicity of the studied populations and the adjustment of confounders. Moreover, in most cohort studies, vitamin C intake was mainly determined by dietary assessments, which are not accurate indicators of the plasma vitamin C concentration (50). Some scholars believe that the discrepant results may be attributable to the pro-oxidative effect of vitamin C, which, despite being an antioxidant, functions as a pro-oxidant in some cases (60, 62). Furthermore, the results of experimental studies differ from those of observational studies. Experimental studies may have tended to include high-risk groups and use high doses of vitamin C. Thus, from the results of these studies, it may not be possible to determine whether long-term low-dose dietary vitamin C intake affects the risk of ischemic stroke in the general population. The discrepant results may also be due to the poor lifestyle habits of participants with low vitamin C intake in cohort studies. Although most studies adjusted for multiple confounders, such as smoking, alcohol consumption, and a history of diabetes or hypertension, they did not control for key dietary confounding factors, such as the intakes of dietary fiber, whole grains, nuts, or salt, which are known to influence the risk of ischemic stroke (63).

## Conclusions

The purpose of this review is to describe the research progress on the relationship between vitamin C and ischemic stroke. As an effective antioxidant, vitamin C plays an important role in reducing the risk of ischemic stroke by protecting the cardiovascular system and preventing atherosclerosis through anti-inflammatory, antioxidant and endothelial protective effects. However, it remains unknown whether the patients with stroke should be administered vitamin C to decrease their level of oxidative stress; whether long-term supplementation of vitamin C is required; what amount of supplementation is optimal; and what is the best source of supplementation. The results of many cohort studies have shown that long-term dietary intake of vitamin C can reduce the risk of ischemic stroke, but the results of the studies so far are not completely consistent; more prospective clinical trials are needed to confirm the role of optimal vitamin C status in stroke management and the effectiveness of this supplementation during stroke.

## Data Availability Statement

The raw data supporting the conclusions of this article will be made available by the authors, without undue reservation.

## Author Contributions

PS and LW conceived the idea for this initiative. XT contributed to reading the literature, preparation of figures and the table, and writing the manuscript. HL and YX assisted with writing and revising the manuscript. All authors read and approved the final manuscript.

## Conflict of Interest

The authors declare that the research was conducted in the absence of any commercial or financial relationships that could be construed as a potential conflict of interest.

## Publisher's Note

All claims expressed in this article are solely those of the authors and do not necessarily represent those of their affiliated organizations, or those of the publisher, the editors and the reviewers. Any product that may be evaluated in this article, or claim that may be made by its manufacturer, is not guaranteed or endorsed by the publisher.
